# Ultrasound assessment of visual loss during severe preeclampsia: a case report

**DOI:** 10.1186/s13089-018-0087-2

**Published:** 2018-02-12

**Authors:** Fehmi Ferhi, Abdeljalil Khlifi, Feten Hachani, Khalil Tarmiz, Khaled Benjazia

**Affiliations:** 1 Department of Anaesthesiology and Critical Care Medicine, Farhat Hached University Hospital Center, 4002 Sousse, Tunisia; 2 The Research Unit on Maternal Morbidity and Mortality UR17SP08, Sousse, Tunisia; 3 Ibn Jazzar Medical School Sousse, Avenue Mohamed El Karoui, 4002 Sousse, Tunisia; 4 Department of Obstetrics and Gynaecology, Farhat Hached University Hospital Center, 4002 Sousse, Tunisia

**Keywords:** Cortical blindness, Ocular ultrasound, Optic nerve sheath diameter, Preeclampsia, Retinal detachments

## Abstract

Bilateral retinal detachments and cortical blindness are rare complications of preeclampsia and the association of the two pathologies is exceptional. We report the case of a preeclamptic patient who presented with an acute bilateral vision loss. Besides, her ocular ultrasound revealed bilateral retinal detachments and an elevated optic nerve sheath diameter. The patient underwent an urgent cesarean section. Subsequently, magnetic resonance imaging and ocular fundus examination confirmed the diagnosis.

## Background

Preeclampsia is a multisystem hypertensive disorder and is mainly responsible for the world’s largest maternal morbidity and mortality. Visual symptoms, such as scotomota, amaurosis, blurred vision, diplopia, chromatopsia, or homonymous hemianopsia, may occur in 25% of preeclamptic women [1]. Blindness is usually attributed to retinal abnormalities including edema and vascular changes, such as retinal arteriolar vasospasm, thrombosis of the central retinal artery, or retinal detachment. Transient cortical blindness due to focal cerebral edema is estimated to occur in about 1–15% of eclamptic women [2].

Given the magnitude of the neurological effects of preeclampsia, real-time assessment of the cerebrovascular and ocular changes associated with this condition are challenging with established modalities such as computed tomography, magnetic resonance imaging and ocular fundus examination [3, 4].

We report the case of a preeclamptic patient who presented with acute bilateral vision loss. Bedside ocular ultrasound revealed a bilateral retinal detachment and an elevated optic nerve sheath diameter.

## Case report

A 24-year-old primigravida woman presented to the obstetric emergency department at 35 weeks of gestation complaining of bilateral vision loss. There was nothing abnormal with her previous medical history and she had no history of previous ophthalmologic disorders. The patient reported a mild headache that has been going on for the past 2 days.

Clinical examination showed an important peripheral edema. Vital signs were as follows: pulse rate 105 beats/min, blood pressure 186/115 mmHg, respiratory rate 20 breaths/min, temperature 36.9 °C, and oxygen saturation 98% on air. Visual acuity in the two eyes was finger-counting at less than 1 m. Her pupils were equal and reactive to light. Neurologic examination showed exaggerated deep tendon reflexes.

Proteinuria dipstick was 3+. Laboratory tests revealed anemia at 103 g/L, thrombocytopenia with platelets count at 97 × 10^9^/L and elevated liver enzymes (GOT 82 U/I, GPT 78 U/I, alkaline phosphatase 168 U/I). The remaining laboratory tests, including electrolytes, creatinine, bilirubin, and coagulation tests, were within normal limits.

A bedside ultrasound was performed by an anesthesiologist trained in bedside focused ultrasonography using a 7.5-MHz linear probe (mindray DC 70 Shenzhen, China). Sagittal and transverse planes of the right and left globe were obtained by asking the patient to close her eyelids and then applying ultrasound gel to the inside and outside of a sterile sheath covering the probe as it was gently placed on each eyelid. To measure the optic nerve sheath diameter (ONSD), the placement of the probe was adjusted to view the entry of the optic nerve into the globe. We used an electronic caliper and an axis perpendicular to the optic nerve 3 mm behind the lamina cribosa. The Mechanical Index and Thermal Index were reduced to 0.2 and 1, respectively.

**Fig. 1:**
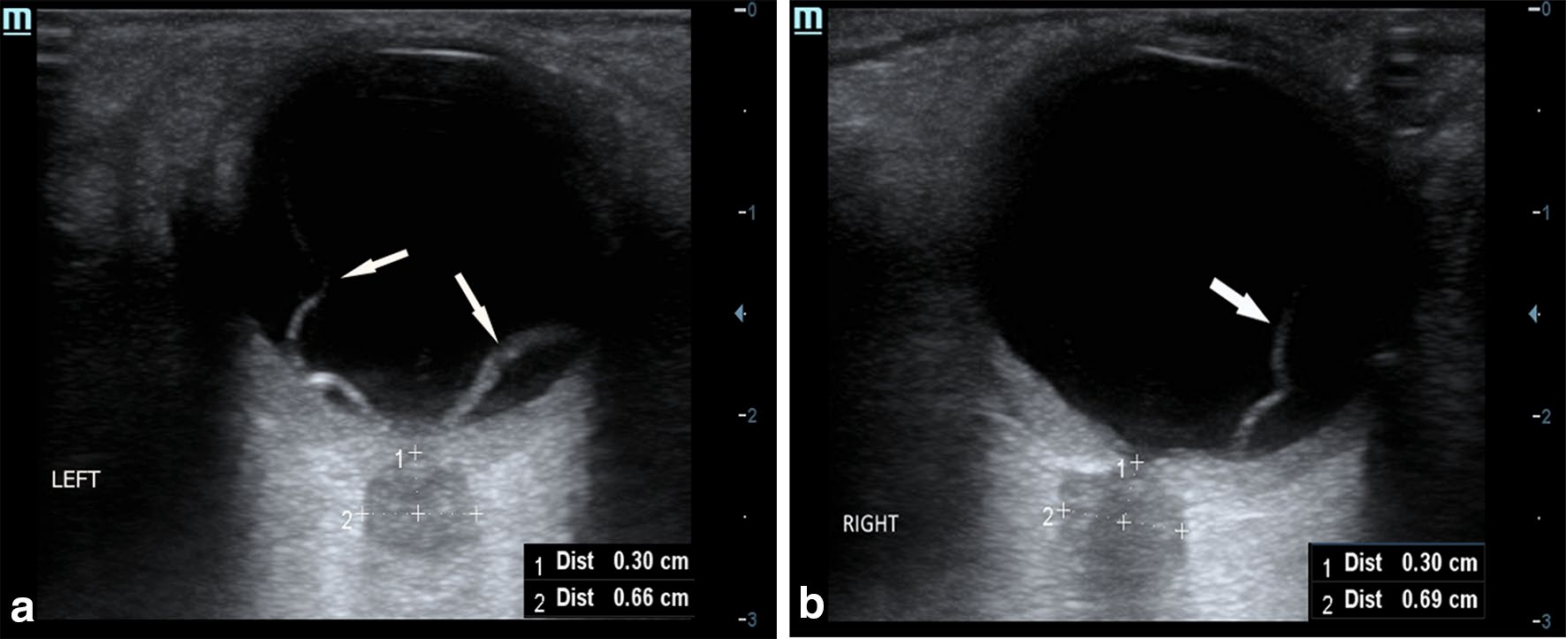
**a** Ocular ultrasound of the left eye demonstrating retinal detachment (white arrows) and optic nerve sheath diameter enlargement. **b** Ocular ultrasound of the right eye demonstrating retinal detachment (white arrow) and optic nerve sheath diameter enlargement

Sonographies detected in the right and left eye a hyper-echoic stripe extending to the optic nerve head, but not across it. The point of fixation of the retina at the optic nerve head was respected. When the patient was also asked to look in various directions with her eyelids closed, the hyper-echoic line undulated with the associated ocular movements, which was suggestive of retinal detachment (RD). The ONSD was measured at 6.9 mm in the right eye (Fig. 1a) and 6.6 mm in the left eye (Fig. 1b), suggesting an elevated intracranial pressure. The patient tolerated the sonogram without pain or complications.

Obstetric ultrasound revealed an intra-uterine fetus with a heart rate of 140 beats/min and a fetal biometry consistent with a 34-week gestation.

Based on these findings, the patient was diagnosed with severe preeclampsia complicated by hemolysis, elevated liver enzymes and low platelets (HELLP) syndrome, bilateral RD and cerebral edema. Therefore, the patient underwent an urgent cesarean section delivery under spinal anesthesia and was immediately administrated an intravenous Magnesium Sulfate and Nicardipine infusion which were maintained 24 h after delivery. Blood pressure was then controlled with Methyldopa 500 mg 6 hourly and Amlodipine 10 mg 12 hourly.

Magnetic resonance imaging (MRI) performed 24 h after delivery showed T2 hyperintensities on flair indicating parieto-occipital and temporal distribution of vasogenic edema. A dilated fundoscopic examination using an ophthalmoscope performed by an ophthalmologist subsequently confirmed the diagnosis of bilateral exudative retinal detachments.

During 1 week, a daily ocular sonogram was performed. The ONSD was back to a normal range after arterial blood pressure control. The RD regressed but it was difficult to objectify it.

Within 3 weeks of delivery, her visual acuity had returned to normal without any further treatment. At that time, the slit lamp examination was normal.

## Discussion

This case highlights a possible association between two etiologies of vision loss during severe preeclampsia and the interest of bedside ocular sonography for reliable assessing.

The most frequent ophthalmologic manifestation of preeclampsia is the vasoconstriction of the retinal arterioles. The prevalence of this disorder increases with severity [5]. It is mostly asymptomatic and regresses without sequelae after delivery. Other clinically noisier manifestations are very rare, and we can mention among them cortical blindness and retinal serous detachment. The association of the two pathologies is exceptional [6].

Computed tomography (CT) and MRI brain imaging are mandatory to make the diagnosis of cerebral edema, but they are expensive and cannot be carried out in real-time assessing [3]. Retinal examination for papilledema or RD seems sometimes difficult. Actually most anesthesiologists and emergency physicians are more comfortable using an ultrasound machine than an ophthalmoscope. In addition, ultrasound is bedside available, reproducible and non-invasive [4].

Preeclampsia can be associated with brain edema and raised intracranial pressure (ICP) [6]. Increases in ICP are transmitted by the cerebrospinal fluid down the perineural subarachnoid space of the optic nerve, causing an expansion of the nerve sheath [7]. ONSD measured by ultrasound has proved to be highly predictive of intracranial pressure in the settings of traumatic brain injury and neurological pathology [8]. Dubost et al. [9] measured ONSD in patients with preeclampsia and compared the findings with those obtained in healthy pregnant women. They found that median ONSD values were significantly higher in preeclamptic patients at delivery and that in about 20% of preeclamptic patients ONSD values were above 5.8 mm. This threshold diameter was associated with 95% risk of raised ICP (above 20 mmHg) [8]. This technique is rapidly acquired by non-radiologist physicians [10, 11].

RD is a well-documented complication of preeclampsia affecting 0.1–2% of patients [12]. Women who have HELLP syndrome are 7 times more likely to develop RD than those who do not have HELLP syndrome. RD is more frequent in primiparous women. It is usually bilateral and tends to be diagnosed during the last trimester or shortly after delivery [13]. The main pathogenesis of this disease includes generalized vasoconstriction and choroidal ischemia affecting the retinal pigment epithelium, leading to a breakdown of the blood–retinal barrier and a leakage of proteins and fluid from the choriocapillaries into the subretinal space [14]. Focal ischemia caused by capillary obstruction is even more severe when associated to hemolysis of the red blood cells, such as the case of HELLP syndrome [13]. Patients usually have complete recovery of vision with complete resolution of RD within 2–12 weeks postpartum [14].

Ocular ultrasound has proved to be a useful tool for diagnosing most ophthalmic pathologies including retinal detachments, vitreous hemorrhage, papilledema, lens dislocation and globe rupture. The sensitivity and specificity of ultrasound for the diagnosis of retinal detachments were reported to be high [15].

Regarding the mechanical and biological risks of exposing the eye to high-frequency probes, sonography side effects have not been reported up to now. In animal studies, rabbit corneas and retinas were exposed to ultrasound ranging from 10 to 60 MHz for up to 30 min with no deleterious effects [16]. Nevertheless, mechanical energy is transmitted into the eye and more than 4–10 × 10^6^ oscillations may warm the intraocular fluid. Therefore, it is important to limit the examination time and gain according to the ALARA (“as low as reasonably applicable”) principle and reduce Mechanical Index and Thermal Index less than 0.23 and 1, respectively.

Actually preeclampsia especially when severe is associated with an increased risk of maternal retinal disease in the decades following pregnancy [17]. Ocular ultrasound may have a useful role in the assessment strategy of neurological and retinal events in preeclampsia, potentially allowing for more appropriately targeted therapy and survey [3].

In this case, ocular ultrasound was performed by an anesthesiologist using a 7.5-MHz linear probe placed gently over the closed eyelid. This rapid bedside examination quickly helped us to refine our diagnosis in a preeclamptic patient with a life-threatening condition and allowed us to make a rapid decision.

## Abbreviations

RD: retinal detachments
ONSD: the optic nerve sheath diameter
HELLP syndrome: hemolysis, elevated liver enzymes, and low platelets syndrome
MRI: magnetic resonance imaging
CT: computed tomography
ICP: intracranial pressure
